# High-Quality GaP(111) Grown by Gas-Source MBE for Photonic Crystals and Advanced Nonlinear Optical Applications

**DOI:** 10.3390/nano15080619

**Published:** 2025-04-18

**Authors:** Karine Hestroffer, Kelley Rivoire, Jelena Vučković, Fariba Hatami

**Affiliations:** 1 Department of Physics, Humboldt-Universität zu Berlin, Newtonstraße 15, 12489 Berlin, Germany; 2 E. L. Ginzton Laboratory, Stanford University, Stanford, CA 94305, USA

**Keywords:** gallium phosphide (111), AlGaP(111), gas-source MBE, photonic crystals

## Abstract

The precise fabrication of semiconductor-based photonic crystals with tailored optical properties is critical for advancing photonic devices. GaP(111) is a material of particular interest due to its high refractive index, wide optical bandgap, and pronounced optical anisotropy, offering unique opportunities for photonic applications. Its near-lattice matching with silicon substrates further facilitates integration with existing silicon-based technologies. In this study, we present the growth of high-quality GaP(111) thin films using gas-source molecular-beam epitaxy (GSMBE), achieving atomically smooth terraces for the homo-epitaxy of GaP(111). We demonstrate the fabrication of photonic crystal cavities from GaP(111), employing AlGaP(111) as a sacrificial layer, and achieve a quality factor of 1200 for the cavity mode with resonance around 1500 nm. This work highlights the potential of GaP(111) for advanced photonic architectures, particularly in applications requiring strong light confinement and nonlinear optical processes, such as second-harmonic and sum-frequency generation.

## 1. Introduction

The field of photonics has witnessed transformative advancements driven by the need for devices that can manipulate and control light with unparalleled precision. Photonic crystals, with their ability to confine and guide light through engineered optical bandgaps, are central to this progress, enabling applications in optical communication, quantum information processing, and nonlinear optics. Achieving these capabilities, however, demands both advanced fabrication techniques and materials with special optical and structural properties.

Gallium phosphide (GaP) has emerged as a standout material for such applications due to its unique combination of characteristics [1]. With a high refractive index (n = 3.5 at 0.5 µm and n = 3 at 6 µm), a wide optical bandgap (2.26 eV, the indirect bandgap at 300 K), and pronounced optical anisotropy, GaP supports strong light confinement and efficient waveguiding. Additionally, its high thermal and mechanical stability makes it robust under demanding operating conditions. Most notably, GaP exhibits high nonlinear optical properties, making it an excellent candidate for frequency conversion processes such as second-harmonic generation (SHG) and sum-frequency generation (SFG) [2,3]. Its high second-order nonlinear susceptibility (χ^(2)^) allows for the efficient generation of new frequencies, which is critical for applications in wavelength conversion, ultrafast optics, and on-chip photonic integration. The third-order nonlinear susceptibility (χ^(3)^) of GaP is notably large, attributed to its two-photon absorption properties and strong Kerr nonlinearity (n_2_ = 1.1 × 10^17^ m^2^/W at 1550 nm), further establishing it as a key material in nonlinear photonics [4]. The broad optical transparency window of GaP, spanning from the visible to the near-infrared, adds to its versatility in a wide range of photonic devices [1].

Another key advantage of GaP is its near-lattice matching with silicon substrates, which facilitates integration into existing silicon-based photonic and electronic platforms. This compatibility enables hybrid systems that combine the scalability of silicon photonics with the unique optical functionalities of GaP.

To date, research has predominantly focused on GaP(001) photonic crystal cavities, utilizing AlGaP(001) as the sacrificial layer (e.g., [1,2,3]). This approach aligns well with monolithic integration into mature and cost-effective Si(001) platforms [5]. However, GaP(111) exhibits higher optical nonlinearity coefficients compared to the (001) orientation [6], making it a superior candidate for applications where performance supersedes cost constraints. Specifically, GaP(111) membranes are promising for lab-scale nonlinear optics, and specialized photonic devices requiring low-loss waveguides or high-efficiency frequency conversion. While GaP(001) remains the pragmatic choice for CMOS-compatible mass production, GaP(111) offers enhanced optical performance for nonlinear photonics applications.

Despite this potential, GaP(111)-based photonic crystals remain underexplored due to challenges in epitaxial growth of high-quality GaP(111) membranes and AlGaP(111) This sacrificial layers.

In this study, we address the challenges of homoepitaxy of GaP(111) and heteroepitaxy of GaP/AlGaP(111) using gas-source molecular-beam epitaxy (GS-MBE). We successfully achieved the growth of atomically smooth GaP(111) layers. By utilizing AlGaP(111) as a sacrificial layer, we demonstrated the fabrication of photonic crystal cavities from GaP(111) and achieved a quality factor (Q) of 1200 for the cavity mode with resonance at 1500 nm. These results are particularly significant for nonlinear optical applications and integrated photonics, where high-quality cavities are essential to enhance nonlinear interactions through tight optical confinement and prolonged light-matter interaction times.

## 2. Materials and Methods

The growth of GaP, AlP, and AlGaP was carried out using a Riber 32P gas-source molecular-beam epitaxy (GSMBE) system (Riber S.A., Bezons, France), equipped with PH_3_ as the phosphorus source and solid Al and Ga sources. The PH_3_ gas was cracked by a high-temperature cracker at 850 °C, with its flux regulated using a flowmeter calibrated with N_2_. The deposition rates of Al and Ga were determined through Reflection High-Energy Electron Diffraction (RHEED) oscillations on GaAs(001) substrates. Substrate temperature was monitored with a pyrometer directed at the surface of the substrate.

Undoped GaP(111)B substrates (ITME, Warsaw, Poland) with an off-orientation of ±0.5° towards <110> were prepared, baked in an Ultra-High Vacuum (UHV) for 60 min at 200 °C, and subsequently transferred into the MBE reactor. Native oxide desorption was performed at 600 °C for 40 min under a PH_3_ flux of 2.7 sccm (standard cubic centimeters per minute). The substrate temperature was then adjusted to the desired growth temperature, after which epitaxy was initiated. The morphological and structural properties of the epitaxial layers were characterized using various techniques, including RHEED, scanning electron microscopy (SEM), and Atomic Force Microscopy (AFM). The alloy composition was measured using X-Ray Diffraction (XRD). The surface reconstructions were monitored in situ using RHEED, and the surface roughness of the final structures was examined with AFM.

To fabricate GaP photonic crystal structures, three-hole linear defect (L3) [7] cavities were designed and implemented in a GaP membrane. This membrane was grown atop a sacrificial Al_x_Ga_1−x_P layer on a (111)B-oriented GaP wafer.

To produce the cavities in the GaP membrane, a 360 nm layer of ZEP520A resist was spun onto the sample, and the photonic crystal structures were patterned using electron-beam lithography. The patterned sample was subsequently dry-etched using reactive ion etching (RIE) with an Ar/BCl_3_/Cl_2_ plasma. To calibrate the dry etch rate for small features in GaP, trenches of varying widths were etched, cleaved, and measured using a scanning electron microscope.

After finishing the processing steps, the excess photoresist was removed with oxygen plasma. The sacrificial AlGaP layer was then undercut using hydrofluoric acid, resulting in suspended membrane structures with high index contrast. For an aluminum content of x = 0.6 in Al_x_Ga_1−x_P, the selectivity of the wet etch process was insufficient to completely remove the sacrificial layer without partially affecting the GaP membrane. Increasing the aluminum content to x > 0.8 resolved this issue by improving the selectivity of the undercut process.

The fabricated photonic crystal resonators were characterized using a confocal cross-polarized reflectivity setup [8]. This technique leverages polarization control to achieve a high signal-to-noise ratio and enables the probing of cavities without internal light sources. A vertically polarized (V) probe beam was directed through a polarizing beam splitter (PBS) and a half-wave plate (HWP) onto the photonic crystal cavity, which supports a vertically polarized mode (V). The reflected output was observed through the PBS, functioning as a horizontal (H) polarizer. When the angle (θ) between the fast axis of the HWP and the vertical direction was set to zero, the cavity-coupled light, reflected with V-polarization, did not transmit through the beam splitter. By rotating the HWP, a portion of the cavity-coupled light was transmitted through the PBS to the output port, with an intensity following a sin(4θ) dependence. A tungsten–halogen lamp served as a broadband input source, and the output field was measured using a spectrometer equipped with a liquid nitrogen-cooled CCD.

## 3. Results and Discussion

### 3.1. Gas-Source MBE of GaP(111)B and AlGaP(111)B

Our study first investigates the influence of substrate temperature and the phosphine flux on the surface reconstruction of the GaP(111)B prior to growth using RHEED.

In general, III-V(111)B surfaces are terminated by a half bilayer of group V atoms, resulting in a phosphorus (P)-rich GaP(111)B surface, particularly after thermal cleaning under a PH_3_ flux. When surface atoms rearrange themselves from their original bulk positions to form a two-dimensional atomic net, surface reconstruction occurs. In situ RHEED measurements provide direct access to surface reconstructions and associated atomic configurations.

The first step before growth is the outgazing process under PH_3_ flux. During this step, no surface reconstruction is observed at any substrate temperature or PH_3_ flux. However, after the growth of just a few nanometers of GaP, a series of four distinct surface reconstructions is consistently observed.

**Figure 1:**
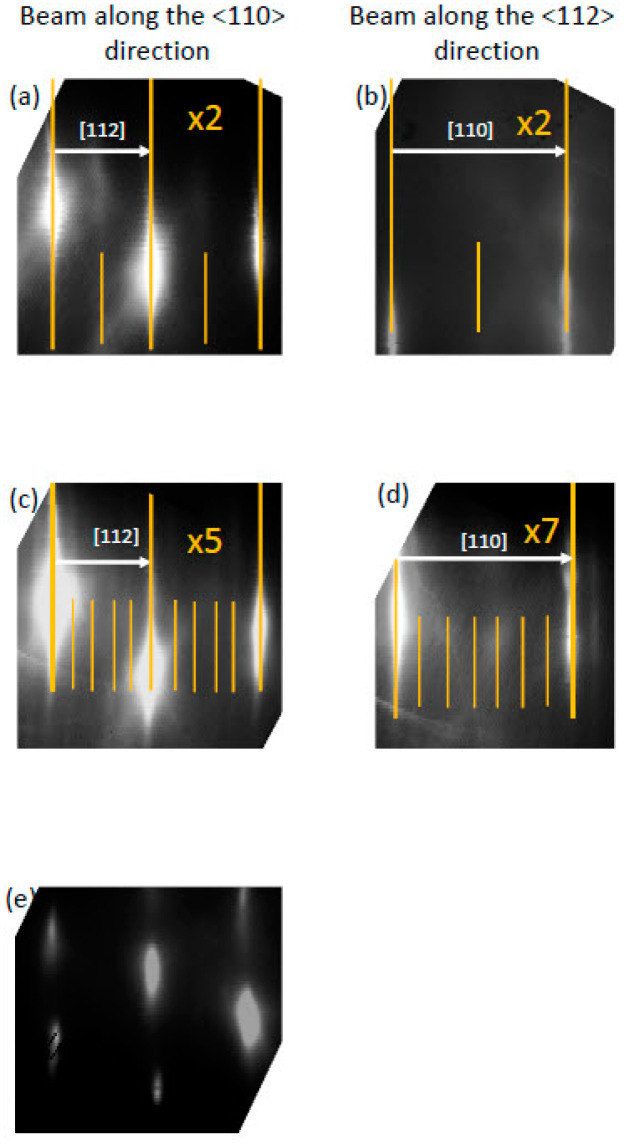
RHEED patterns of the GaP(111)B surface at different growth temperatures under fixed PH_3_ flow, exhibiting distinct surface reconstructions: (**a**,**b**) a (2 × 2) pattern at lower temperature; (**c**,**d**) a (√19 × √19) pattern at higher temperature; and (**e**) a broken (1 × 1) at an even higher temperature.

The temperature of the substrate was first increased from 500 to 650 °C while keeping a fixed PH_3_ flux of 3.5 sccm. The temperature-dependent evolution of the RHEED patterns is illustrated in Figure 1. At the lowest temperatures between 500 °C and ~520 °C, bright sharp main streaks were observed together with additional streaks with weaker intensity. The weaker streaks have a ×2 periodicity along both <110> and <112> azimuths (Figure 1a,b). Increasing the temperature to about 525 °C leads to a quick transition during which the ×2-periodic streaks completely fade away. At higher temperatures up to 540 °C, new weak-intensity streaks appear with ×5 and ×7 periodicity along the <110> and <112> azimuths, respectively (Figure 1c,d). Upon further increasing the temperature above 610 °C, the weak reconstruction strikes first disappear and then the main strikes break down (Figure 1e), suggesting that the surface starts decomposing at such high temperatures.

The observed RHEED patterns are consistent with those reported for the GaAs(111) surfaces, with the ×2 and ×2 periodic patterns along the <110> and <112> azimuths being identified as a (2 × 2) surface reconstruction, and the ×5 and ×7 periodic patterns along the <110> and <112> azimuths as a (√19 × √19) surface reconstruction [9].

**Figure 2:**
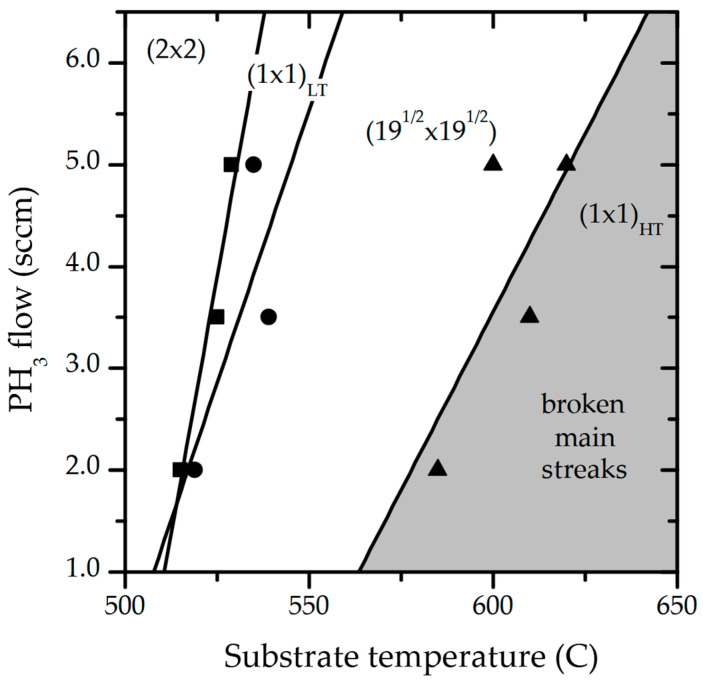
The summary of the static surface reconstruction of GaP(111)B as a function of substrate temperature and phosphine flow. The square, circle, and triangle symbols represent the observed RHEED pattern transitions. The lines indicate the general trends and are provided as a guide.

In order to further investigate the static surface reconstruction, the measurements were performed at different phosphine flows at the given temperature. Figure 2 presents a diagram which summarizes the surface reconstruction transition’s dependence on the substrate temperature and the PH_3_ flow.

Our data indicate that, regardless of the PH₃ flow, a (2 × 2) surface reconstruction is observed at lower temperatures, while a (√19 × √19) reconstruction appears at higher substrate temperatures. However, with increasing PH₃ flow, an intermediate phase emerges between the (2 × 2) and (√19 × √19) reconstructions. This phase, which we identify as a non-reconstructed surface, is referred to as (1 × 1)_LT_ (LT: low temperature), as illustrated in Figure 2. As the substrate temperature rises, the (1 × 1)_LT_ pattern transitions to the (√19 × √19)-reconstructed surface. Further increasing the temperature causes the (√19 × √19) reconstruction streaks to vanish, while the main streaks break down, suggesting surface decomposition at these elevated temperatures. We refer to this state as (1 × 1)_HT_ (HT: high temperature). It is noteworthy that the temperature region of (1 × 1)_LT_ transition is very narrow and less than 20 °C, and it expands slightly with increasing PH_3_ flow.

Few studies have reported on GaP(111)B surface reconstructions, primarily based on scanning tunneling microscopy (STM) and low-energy electron diffraction (LEED) [10,11,12,13]. An unreconstructed (1 × 1) surface was reported at temperatures above 250° [12]. We previously reported a (3 × 3)-reconstructed surface during GSMBE [14]. This reconstructed surface can be a stable and dominant phase during the homoepitaxy of GaP(111)B under a very low III/V ratio and was observed for Ga deposition rates lower than 0.2 ML/s [14].

Earlier work on the epitaxy of GaAs(111) established that the deterioration of the (2 × 2) surface reconstruction is related to insufficient group V element coverage. The surface reconstructions and corresponding RHEED patterns are similar to those reported on GaAs(111)B [15,16,17,18]. The intermediate phase between (2 × 2) and (√19 × √19), which we refer to as (1 × 1)_LT_ and identify as a non-reconstructed surface, is also reported for GaAs(111) [16,17].

Increasing the PH_3_ flow shifts the transition temperature of a given surface reconstruction to higher values, a phenomenon also reported for the growth of GaAs(111) [15,16,17]. This indicates that the type of GaP(111)B surface reconstruction depends on phosphorus coverage. Specifically, (2 × 2)-reconstructed surfaces correspond to higher P coverages, as illustrated in Figure 2.

**Figure 3:**
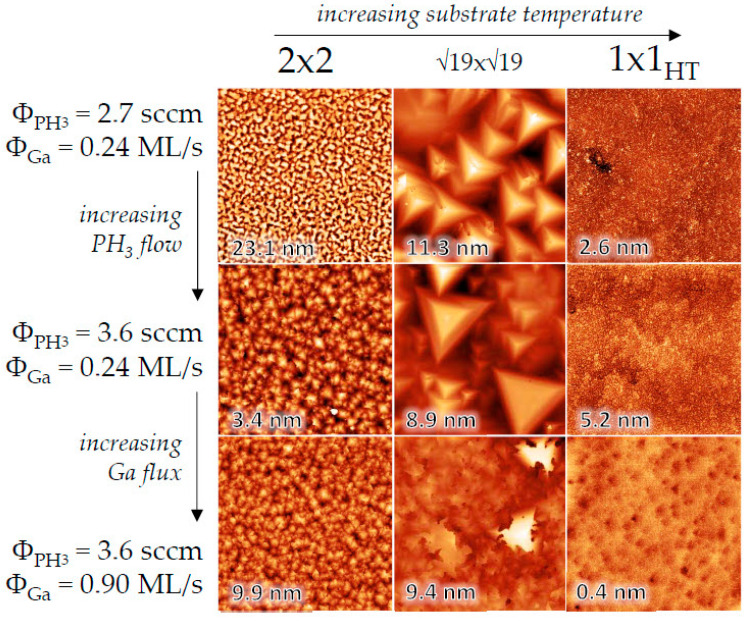
10 × 10 µm^2^ AFM images of GaP films grown at different substrate temperatures and different Ga and PH_3_ fluxes. The color scale varies for each image in order to emphasize the details of the characteristic features. The root-mean-square surface roughness is indicated on the images.

We further investigated the surface properties of homoepitaxial GaP grown on the different reconstructions described above. Figure 3 shows 10 × 10 µm^2^ AFM images of GaP films grown under varying substrate temperatures, PH_3_ flow, and Ga flux. The color scale is adjusted individually for each image to highlight the characteristic surface features. The root mean square (RMS) surface roughness is noted on each image, and black arrows indicate increases in substrate temperature, PH_3_ flow, or Ga flux.

The surface roughness of GaP films shows clear correlations with both growth temperature and initial surface reconstruction, as demonstrated in Figure 3. Low-temperature growth on the (2 × 2)-reconstructed surface consistently produces grainy surface morphologies, while growth on the (√19 × √19)-reconstructed surface leads to distinct faceted pyramid formation. In contrast, high-temperature growth on the (1 × 1)_HT_ surface reliably yields atomically smooth films. While comprehensive theoretical simulations would be required to fully explain the mechanistic relationship between surface reconstructions and roughness evolution, we propose that three key factors dominate the observed behavior: stoichiometric balance, hydrogen passivation from thermally cracked PH₃ during GSMBE growth, and adatom surface kinetics.

For the (2 × 2) reconstruction, insufficient PH₃ flux promotes phosphorus vacancy formation and gallium adatom accumulation, resulting in high surface roughness with RMS values exceeding 20 nm. When PH₃ flux is optimized, the resulting stoichiometric balance suppresses these defects to achieve remarkably smooth surfaces with RMS roughness around 3 nm. However, when gallium flux becomes excessive, gallium-rich domains form, resulting in an intermediate roughness of approximately 10 nm RMS. The more complex (√19 × √19) reconstruction, characterized by its mixed gallium/phosphorus adatom arrangement and intricate stacking configuration, displays higher roughness that remains relatively constant across the range of flux conditions we examined.

The (1 × 1)_HT_ surface achieves minimal roughness, when grown under high but balanced PH_3_ and gallium fluxes. In these conditions, hydrogen from the cracked PH_3_ precursor effectively passivates surface dangling bonds, mimicking intentional surface termination approaches. This equilibrium state successfully suppresses both vacancy formation and adatom clustering. We observe that exceeding the optimal PH₃ flux level (increasing from 2.7 to 3.6 sccm) while maintaining a low gallium deposition rate of 0.24 ML/s increases surface roughness, likely due to gallium starvation caused by phosphorus overpopulation.

Scanning tunneling microscopy and low-energy electron diffraction studies have previously characterized pyramid formation on GaP(111)B, demonstrating that these features exhibit {-1-10} side facets and decrease in size during annealing processes [19]. The {-110} family of planes in GaP contain equal numbers of gallium and phosphorus atoms and, given the relatively weak ionicity of GaP compared to other III-V materials, are expected to remain charge-neutral without requiring surface reconstruction [20]. The pyramids observed alongside step edges during growth at the highest temperatures within the (1 × 1)_HT_ window may consist of pure metallic gallium resulting from the thermal decomposition of GaP [21]. Growth occurring just above the (√19 × √19) → (1 × 1)_HT_ transition temperature appears to be most favorable, promoting the step-flow growth mode without pyramids.

These findings align with previous reports for GaAs(111)B surfaces, where growth within different reconstruction windows produces distinct morphological outcomes [15,16,17,18]. However, for GaAs(1-11)B, pyramid formation occurs when growth is performed within the temperature and arsenic flux range corresponding to the (2 × 2) reconstruction window or its transition region, while specular surfaces form in the (√19 × √19) reconstruction window [18].

**Figure 4:**
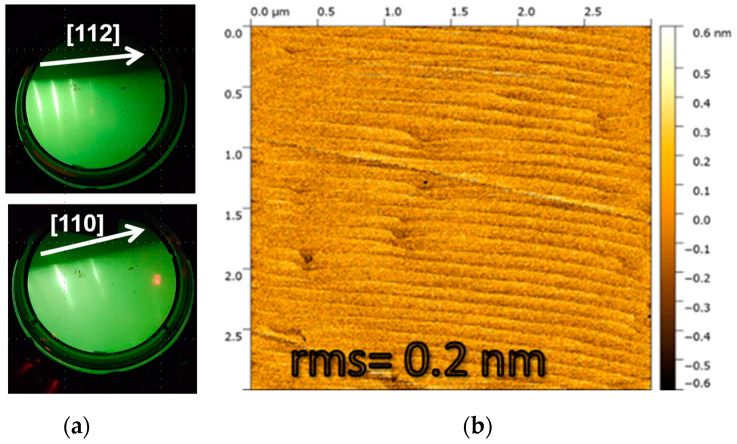
(**a**) RHEED patterns reordered with the primary electron beam aligned along the [112] and [110] azimuths; (**b**) step-flow growth mode on the (1 × 1)_HT_ surface. The AFM image shows a 3 × 3 μm^2^; area with an rms roughness of 0.2 nm. Terraces are ~150 nm wide and 1 ML high.

Our results clearly demonstrate that homoepitaxial growth in the (1 × 1)_HT_ regime leads to the smoothest surfaces, with roughness values in the angstrom range. At a high growth temperature, just before the sharp lines of the (1 × 1)_HT_ RHEED pattern began to degrade, we achieved step-flow growth. This mode yielded a root-mean-square roughness of 0.2 nm, with terraces approximately 150 nm wide and 1 monolayer (ML) high. Figure 4a displays the RHEED patterns recorded during the homoepitaxy of such a GaP(111)B layer, under a PH_3_ flux of 3.6 sccm and a Ga deposition rate of 1.5 ML/s. The images show the patterns with the primary beam aligned along the [112] and [110] azimuths. An ex situ AFM image of the sample after growth is also presented in Figure 4b.

After optimizing the growth conditions for GaP(111)B, we used these parameters to grow an atomically smooth buffer layer for the epitaxy of Al_x_Ga_1−x_P, with aluminum content (x) ranging between 0.6 and 1. Our investigation revealed that the optimal growth temperature for AlGaP(111)B is significantly lower than for GaP. The best AlP layer, with an rms roughness of approximately 1 nm, was grown at 490 °C under a PH_3_ flux of 2.0 sccm and a total growth rate of 1.5 ML/s. The corresponding RHEED pattern appeared sharp and (1 × 1).

**Figure 5:**
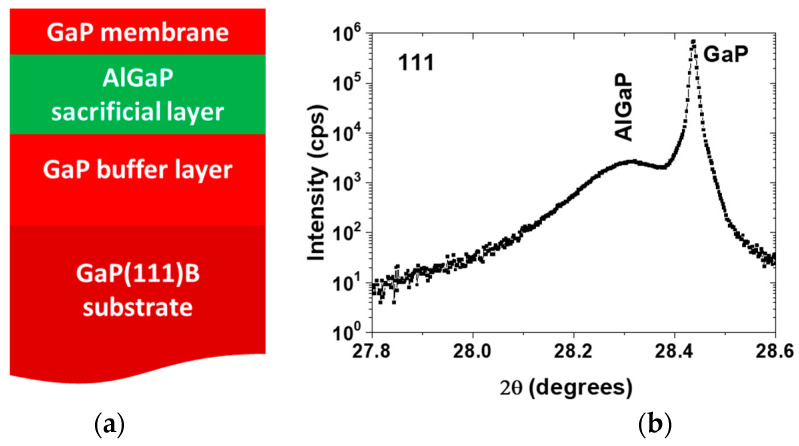
(**a**) A schematic cross-sectional view of the structure, grown for the photonic crystal; (**b**) an exemplary XRD spectrum of one of the AlGaP/GaP(111)B structures.

For AlGaP samples with lower aluminum content, slight adjustments were made to the temperature and PH₃ flux. To prepare the structure for photonic crystal fabrication, GaP layers with thickness between 150 and 250 nm were grown on top of the AlGaP layer at 560 °C. Figure 5a presents a schematic cross-sectional view of the structure, while Figure 5b displays a representative XRD spectrum.

### 3.2. Fabrication and Examination of the Photonic Crystal Cavities

The epitaxial structures then were fabricated using the steps described in the last section to create a GaP(111) photonic crystal resonator, featuring an L3 cavity. The photonic crystal cavities incorporated a perturbation design to enhance the coupling efficiency between the cavity and the objective lens. We used here a 160 nm thick GaP membrane. This membrane was grown atop a 700 nm thick sacrificial AlGaP layer. The photonic crystal structure featured a lattice constant of a = 500–560 nm, a hole radius of r/a ≈ 0.2–0.25, and a slab thickness of d/a ≈ 0.3. For slightly modified cavity designs, coupling efficiency could be further improved by perturbing the photonic crystal structure, as detailed in reference [22].

**Figure 6:**
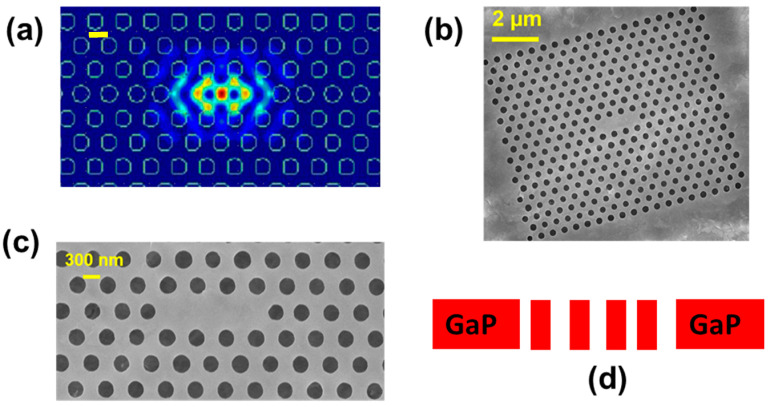
(**a**) Finite-difference time-domain simulation of the electric-field intensity inside the cavity (scale bar: 300 nm); (**b**,**c**) plan-view SEM images of the L3 photonic crystal cavity, where black circles represent air holes and the gray area corresponds to the GaP layer. (**d**) A schematic of the side view of the fabricated GaP membrane.

The cavities were examined using scanning electron microscopy and reflectivity measurements. The electric-field profile of the fundamental L3 cavity mode is depicted in Figure 6a. Figure 6b,c present the SEM images of the L3 GaP photonic crystal cavity, where black circles represent air holes fabricated via dry etching, and the gray area corresponds to the GaP layer. The side view of the fabricated GaP membrane is shown schematically in Figure 6d.

**Figure 7:**
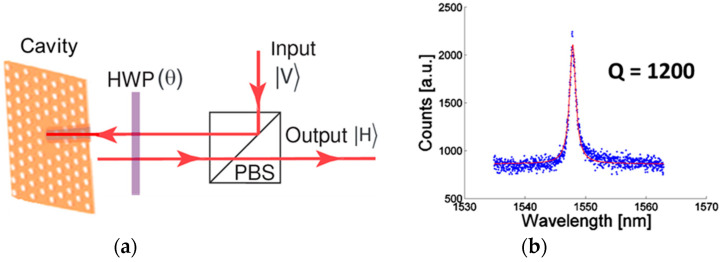
(**a**) The experimental setup using a PBS and a HWP. The cavity signal observed at the output follows a sin(4θ) dependence where theta is the HWP setting (θ = 0 corresponds to V polarization). (**b**) The cavity resonance at 1500 nm was measured at room temperature with the configuration of (**a**). Fitting to a Lorentzian (solid line) gives Q = 1200.

Using a confocal cross-polarized reflectivity measurement setup (Figure 7a), we probed the fabricated resonator. The cavity signal exhibited a sin(4θ) dependence, where θ is the half-wave plate (HWP) setting (θ = 0 corresponds to V polarization). The cavity resonance was measured at ~1500 nm at room temperature, with a Lorentzian fit yielding a quality factor (Q) of approximately 1200 (Figure 7b).

Building on the optimized epitaxy conditions used to grow GaP/AlGaP structures for fabricating the L3 photonic crystal cavity, we aimed to approach the theoretically predicted quality factor. However, the experimentally measured value of 1200 is about an order of magnitude lower, due to imperfections introduced during growth and fabrication. To achieve the predicted quality factors, a pristine fabrication process and crystallographically perfect GaP membrane are essential to minimize optical losses [23]. The structural perfection of the GaP membrane critically depends on the quality of all three constituent layers and their interfaces: (1) the GaP buffer layer, (2) the AlGaP sacrificial layer, and (3) the top GaP membrane layer. Fabrication imperfections—including variations in hole diameter and position, sidewall roughness, and surface irregularities—degrade photonic crystal performance by introducing optical scattering and disrupting the photonic bandgap. These defects induce light leakage and resonant-mode broadening, substantially reducing the quality factor. In our system, even nanometer-scale sidewall roughness (2–5 nm RMS) or slight hole variations (r/a ≈ 0.2–0.25) can degrade Q by 1–2 orders of magnitude through disrupted optical confinement [23,24].

Despite the discrepancy between achieved and predicted values, the measured Q factor of 1200 still enables a significant Purcell effect. Further improvements in fabrication and design optimization should lead to structures with higher Q.

## 4. Conclusions

In summary, we have demonstrated the growth of high-quality GaP(111) thin films via GSMBE and the fabrication of photonic crystal cavities with a quality factor of 1200. While this value remains below theoretical predictions, it still supports strong light confinement and significant Purcell enhancement. The result highlights the critical dependence of high-Q performance on both membrane quality and precise photonic crystal fabrication. Given the favorable optical properties of GaP(111), this work underscores its potential for advanced photonic architectures, particularly in nonlinear optics and cavity quantum electrodynamics. To fully realize this potential, future work must address remaining challenges in epitaxial growth and nanofabrication to push GaP(111)-based systems toward their fundamental performance limits.

## Data Availability

Data are contained within the article.

## Abbreviations

The following abbreviations are used in this manuscript:

GS-MBE	Gas-Source Molecular-Beam Epitaxy
RHEED	Reflection High-Energy Electron Diffraction
UHV	Ultra-High Vacuum
SCCM	Standard Cubic Centimeters per Minute
SEM	Scanning Electron Microscopy
AFM	Atomic Force Microscopy
XRD	X-Ray Diffraction
LEED	Low-Energy Electron Diffraction
STM	Scanning Tunneling Microscopy
ML	Monolayer
RMS	Root mean square
L3	Three-hole linear defect
RIE	Reactive Ion Etching
PBS	Polarizing Beam Splitter
HWP	half-wave plate
V	vertically polarized mode
H	horizontally polarized mode
Q	Quality Factor
